# Cathepsin B-cleavable doxorubicin prodrugs for targeted cancer therapy

**DOI:** 10.3892/ijo.2012.1754

**Published:** 2012-12-28

**Authors:** YAN-JUN ZHONG, LI-HUA SHAO, YAN LI

**Affiliations:** 1 Department of Oncology, Zhongnan Hospital of Wuhan University; Wuhan 430071, P.R. China; 2 Hubei Key Laboratory of Tumor Biological Behaviors and Hubei Cancer Clinical Study Center, Wuhan 430071, P.R. China

**Keywords:** cancer chemotherapy, cathepsin B, doxorubicin, prodrugs, targeted therapy

## Abstract

Doxorubicin (DOX) is one of the most effective cytotoxic anticancer drugs used for the treatment of hematological malignancies, as well as a broad range of solid tumors. However, the clinical applications of this drug have long been limited due to its severe dose-dependent toxicities. Therefore, DOX derivatives and analogs have been developed to address this issue. A type of DOX prodrug, cleaved by cathepsin B (Cat B), which is highly upregulated in malignant tumors and premalignant lesions, has been developed to achieve a higher DOX concentration in tumor tissue and a lower concentration in normal tissue, so as to enhance the efficacy and reduce toxicity to normal cells. In this review, we focused on Cat B-cleavable DOX prodrugs and discussed the efficacy of these prodrugs, demonstrated by preclinical and clinical developments.

## Contents

1. Introduction
2. Prodrug strategies in cancer treatment
3. Cathepsin B (Cat B) as a prodrug-activating enzyme
4. Cat B-cleavable DOX prodrugs
5. Conclusions

## 1. Introduction

**Figure 1.**
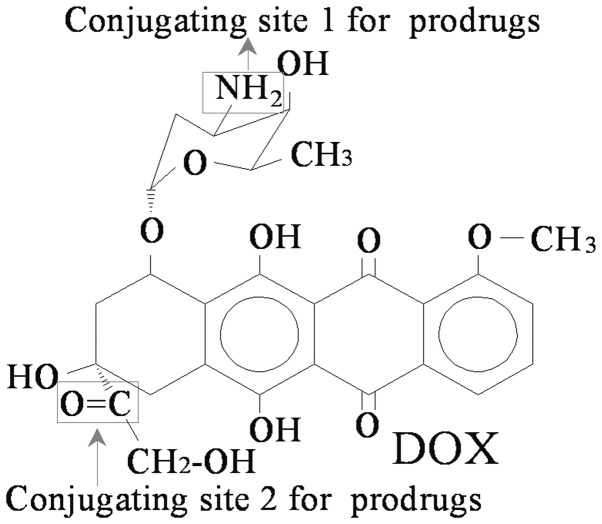
Sructure of DOX. DOX contains an amino group (-NH_2_) on the sixmembered ring, which can conjugate with a carboxyl group (-COOH), and a carbonyl group (-C═O) on another six-membered ring which can react with amino groups. These are the two most common conjugating sites for prodrug design.

Chemotherapy is a major therapeutic approach for the treatment of cancer. Doxorubicin (DOX; Fig. 1), an anthracycline isolated from *Streptomyces* strains, is one of the most effective anticancer drugs used for the treatment of hematological malignancies and a broad range of solid tumors, including lymphoma, Kaposi’s sarcoma, bone tumors, as well as stomach, breast and ovarian cancers (1,2). DOX in its salt form is readily distributed into almost all tissues and intracellular compartments via passive diffusion or active transport following intravenous administration, resulting in indiscriminative toxic effects on all cells exposed to it. Therefore, the clinical application of DOX is limited by its dose-dependent side-effects, such as bone marrow toxicity, cardiotoxicity, nephrotoxicity and hepatotoxicity.

To reduce the side-effects of this drug, significant efforts have been made to develop DOX derivatives and analogs with less toxic effects and improved pharmacological properties. Several strategies have been investigated in clinical and preclinical trials, including various methods of administration, combinations with other chemotherapeutic drugs [e.g., adriamycin, bleomycin, vinblastine and dacarbazine (ABVD), cyclophosphamide, hydroxydaunomycin, oncovin and prednisone (CHOP)] (3), the addition of antioxidant nutrients (4) and cardioprotectors (5–7), the development of liposomes (8) and nanoparticles (9), the effects of acute exercise (10) and the development of prodrugs (11–13). In this review, we focused on the DOX prodrug strategies.

## 2. Prodrug strategies in cancer treatment

Prodrugs are derivatives of drugs which remain inactive in their prototype form but are metabolized in the body to generate the active drugs at the site of action. They are particularly useful in the development of novel antitumor chemotherapeutic drugs, leading to reduced toxicity, improved specificity and the avoidance of multidrug resistance (14,15). The use of prodrugs for targeted therapy is usually based on tumor-associated cell surface markers, such as antigens or receptors, whose expression differs between normal and cancer cells (16,17). Several prodrug strategies have been pursued, including active and passive targeting approaches with antibodies, serum proteins, liposomes and synthetic polymers (18–22). There have been some classic and clinically successful prodrugs, such as capecitabine, an enzyme-activated prodrug, which is converted into 5-fluoro uridine or 5-fluoro-2-deoxyuridine in tumor cells to achieve targeted cytotoxicity (23).

Prodrugs can be divided into high- and low-molecular weight drugs in terms of molecular weight (Mw). The former are internalized by passive or active endocytosis and ultimately become localized in the lysosomal components of cells, while the latter usually enter cells mainly by diffusion (24). The Mw and biodistribution of drugs have important impacts on antitumor efficacy. Macromolecular drugs accumulate in tumor tissues due to the enhanced permeability and retention effect (25–27). A Mw below the renal threshold (∼50,000 g/mol) is rapidly lost from the circulation; therefore, macro-molecular weight drugs may have increased intravascular half-lives, resulting in an increased therapeutic efficacy (27). N-(2-hydroxypropyl) methacrylamide (HPMA), known as one of the most widely used prototypic polymeric drug carriers, was first used to synthesize polymeric drugs in the 1970s, due to its non-immunogenic and non-toxic properties and long circulating half-life (28,29). It has been demonstrated that an HPMA-copolymer Mw of 200,000 to 600,000 g/mol is desirable for the efficient passive targeting of solid tumors (30). Prodrugs bearing HPMA have been developed in preclinical studies and include caplostatin (31,32), P-GDM (33,34) and P-HYD-IgG (35), as well as in phase I/II clinical studies and included HPMA copolymer-Gly-Phe-Leu-Gly-doxorubicin (PK1) (36–39), galactosamine-targeted poly(HPMA)-doxorubicin (PK2) (40–42), PK3 (36), PNU166945 (43), AP5346 (44–48) and AP5280 (49–51).

## 3. Cathepsin B (Cat B) as a prodrug-activating enzyme

Some tumor-associated enzymes, such as proteases, glucuronidases or carboxylesterases, expressed intra- or extracellularly in cancer cells, can release or activate prodrugs. Cat B, a lysosomal cysteine protease in normal cells and tissues, is considered to be one of the best examples of intracellular proteases. It is highly upregulated in malignant tumors and premalignant lesions at the mRNA and protein levels (52). Cat B is localized in perinuclear vesicles, presumably lysosomes in normal cells. However, in tumor cells and oncogene-transformed cells, Cat B is localized in perinuclear vesicles and vesicles throughout the cytoplasm and at the cell periphery (53). Pericellular Cat B participates in degrading processes associated with tumor proliferation, invasion and metastasis. Moreover, exposure to DOX can induce a time- and dose-dependent upregulation of Cat B expression at the mRNA and protein levels (5).

Cat B cleaves Leu, Arg-Arg, Ala-Leu, Phe-Arg, Phe-Lys, Ala-Phe-Lys, Gly-Leu-Phe-Gly, Gly-Phe-Leu-Gly and Ala-Leu-Ala-Leu (18,54–58). There are several low- and high-Mw DOX prodrugs that can be activated by Cat B. Furthermore, DOX immunoconjugates, in which DOX is linked to a carcinoma-specific antibody through Cat B-cleavable oligopeptides, have also been designed (59). All of these conjugates have shown rapid and almost quantitative DOX release in the presence of Cat B. The rate of DOX release depends on the length and structure of the spacer. The tetrapeptide, Gly-Phe-Leu-Gly, has been found to be one of the most suitable spacers. In this regard, the steric interaction between the peptide substrate and Cat B has a significant impact on the release of DOX from prodrugs (60). Therefore, to decrease the steric interaction, it is necessary to integrate a self-immolative spacer, such as para-aminobenzyloxycarbonyl (PABC) between the drug and the oligopeptide substrate.

## 4. Cat B-cleavable DOX prodrugs

**Figure 2.**
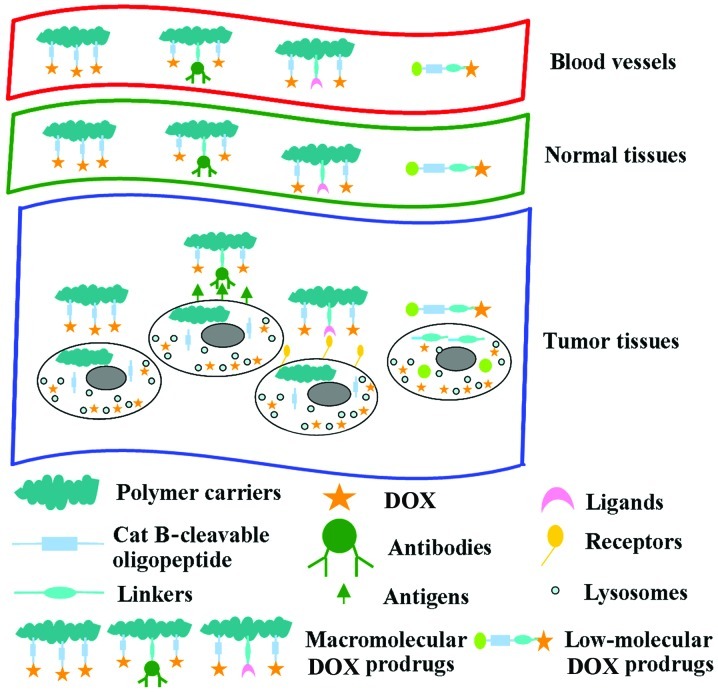
Examples of DOX prodrugs. Macromolecular DOX prodrugs are conjugated with polymer carriers, oligopeptides, with or without antibodies, which can combine with antigens located on the tumor cell surface and ligands that can be recognized by receptors on the tumor cell surface. Low molecular weight DOX prodrugs are combined with oligopeptides, with or without antibodies and ligands. DOX prodrugs remain inactive in blood vessels and normal tissues, but are cleaved by Cat B in tumor tissues and tumor cells, releasing free DOX, which results in targeted cytotoxicity. DOX, doxorubicin; Cat B, cathepsin B.

Examples of Cat B-cleavable DOX prodrugs are illustrated in Fig. 2 and summarized in Table I.

### DOX prodrugs containing the tetrapeptide Gly-Phe-Leu-Gly

The tetrapeptide, Gly-Phe-Leu-Gly, has been proven to be the most effective with respect to both plasma stability and rapid hydrolysis in the presence of Cat B. Therefore, many DOX prodrugs are based on this tetrapeptide.

### PK1

**Figure 3.**
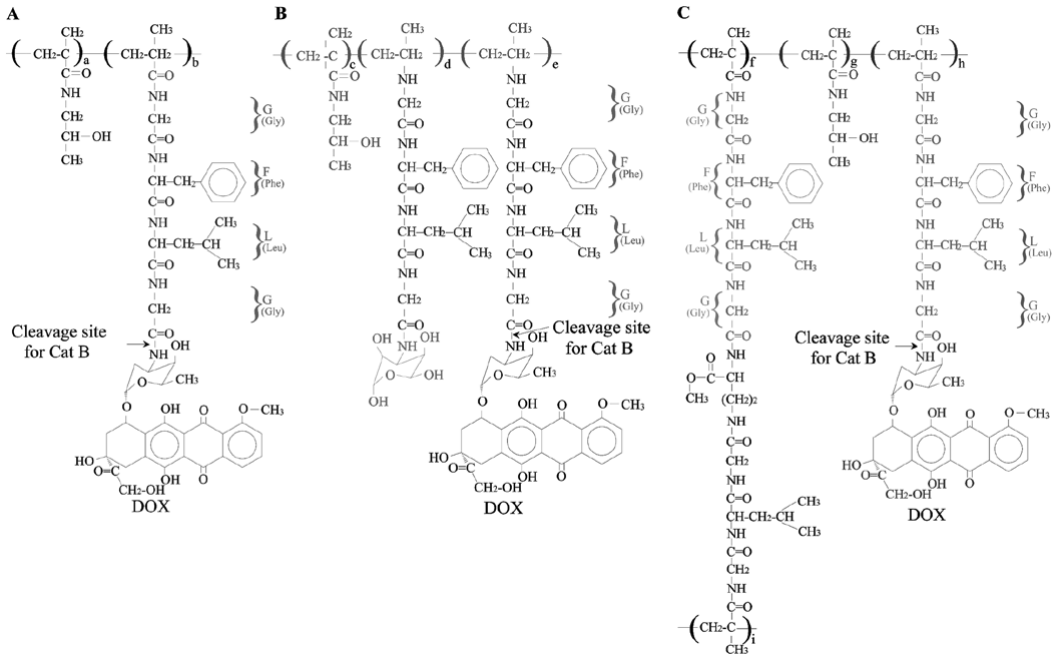
General chemical structures of (A) PK1, (B) PK2 and (C) P-DOX, in which DOX is complexed with (2-hydroxypropyl) methacrylamide (HPMA), by the tetrapeptide linker, Gly-Phe-Leu-Gly (GFLG).

PK1 [FCE28068; P(GFLG)-ADR; DOX-HPMA; doxorubicin-HPMA copolymer conjugate; HPMA-doxorubicin, 8 wt% DOX; Fig. 3A], a polymeric prodrug of Mw ∼30,000 g/mol, was the first macromolecular prodrug to enter clinical trials, and has reached phase II clinical trials.

Preclinical studies using tumor cells, including L1210 leukemia (61–64), A2780 and DOX-resistant A2780/AD ovarian carcinoma cells, have shown that PK1 can partially avoid the ATP-driven P-glycoprotein (Pgp) efflux pump compared with free DOX (65–67). The IC_50_ doses of free DOX and PK1 account for the differences in the mechanisms of cellular uptake (65). In preclinical studies using animal models, including B16F10 melanoma, L1210 leukemia, M5076, LS174T human colorectal xenografts (64) and sensitive and resistant human ovarian carcinoma models (68), PK1 has shown enhanced efficacy. The release of DOX from PK1 *in vitro* and *in vivo* using HPLC analysis has shown only a single peak, representing DOX (64). PK1 does not release DOX in the plasma and the covalently-bound drug is biologically inactive following intravenous administration.

Phase I clinical studies on patients with solid tumors, including colorectal, breast, biliary tract, pancreatic, urinary tract, head/neck, non-small cell lung (NSCL), mesothelioma and stomach cancers, have shown that the maximum tolerated dose (MTD) for PK1 is 320 mg/m^2^, which is 4- to 5-fold higher than the usual clinical dose of free DOX (60–80 mg/m^2^) (69). PK1 decreases non-specific organ toxicities by several folds and allows the active drug to be delivered intracellularly, while maintaining antitumor activity (36,39). Phase II studies using PK1 have shown decreased toxicity with evident activity in breast, NSCL and colorectal cancers. Furthermore, SPECT and γ-camera imaging with ^123^I-labelled drugs have shown obvious tumor accumulation in two metastatic breast cancers (38). PK1 and free DOX greatly differ in their antiproliferative effects and cell death signals in EL-4 cancer cells; treatment with free DOX greatly increases p38 phosphorylation, while PK1 increases it only slightly; PK1 also significantly increases ERK phosphorylation, while free DOX slightly decreases it (70).

In addition, polymer-directed enzyme prodrug therapy (PDEPT) combining HPMA copolymer-Cat B and PK1 has shown activity against a COR-L23 xenograft, whereas PK1 alone has not and in B16F10 melanoma tumors PDEPT has been shown to more effective than either PK1 or free DOX alone (71).

### PK2

PK2 (FCE28069, 27,000 g/mol, 8 wt% DOX; Fig. 3B), the only targeted polymer conjugate containing galactosamine to enter clinical trials, is designed to target the asialoglycoprotein receptor (ASGPR) which is selectively expressed in hepatocytes and hepatoma cell lines (40,72). Pharmacokinetic studies using PK2 in rats and mice have shown effective liver targeting with >70% of the released DOX being selectively targeted to the liver following intravenous administration (73,74). Preclinical studies using rats have demonstrated that PK2 displays a ∼5-fold reduction in cardiotoxicity as opposed to free DOX following intravenous or intraperitoneal administration at various doses (11,64). Furthermore, antitumor activity has been shown to be improved in rodent tumor models (69).

In a pivotal study on a patient with multifocal hepato-cellular carcinoma, HPLC and ^123^I-based imaging showed the biphasic clearance of PK2 from the plasma (half-life, 78±1 and 990±15 min) and ∼30% of the delivered drug accumulated in the liver at 24 h. Moreover, SPECT analysis showed that the radioactivity concentration was 3- to 4-fold higher in peritu-moral liver tissue than in the tumor tissue itself (40).

Phase I/II trials have shown that the MTD of PK2 is 160 mg/m^2^ (DOX equivalent) and several hepatocellular carcinoma patients have displayed partial responses and/or stable disease (41). γ-camera imaging and CT scanning have revealed that 15–20% of total PK2 is retained in the liver and is mostly concentrated in normal liver tissue (normal versus tumor tissue, 5:1), suggesting that the galactosamine-targeted polymer is mainly delivered to normal regions of the liver due to the increased ASGPR expression in the normal liver (75) and the phagocytosis by Kupffer cells with ‘galactose particle’ receptor expression (76). Despite this disparity in PK2 distribution, the drug concentration in tumor tissue was still 12- to 50-fold higher than it would have been with the administration of free DOX alone.

### HPMA copolymer-doxorubicin conjugates (P-DOX)

P-DOX conjugates (Fig. 3C) (77,78) contain the oligopeptide Gly-Phe-Leu-Gly and the N^2^,N^5^-bis(N-methacryloyl-glycylphenylalanyl-leucyl-glycyl) ornithine cross-linker, which permits the synthesis of P-DOX conjugates with various Mws, from 22 to 1230 kDa. The clearance rate of P-DOX from the blood is Mw-dependent and is much slower than that of free DOX (26,77). The therapeutic efficacy has been shown to increase as the Mw of P-DOX increases in nude mice bearing subcutaneous OVCAR-3 xenografts. The low residual concentration of P-DOX in tissues (apart from tumors) helps to avoid potential long-term side-effects (77). The toxicity against hematopoietic precursors and normal lymphocytes of inbred mice is considerably decreased (78).

### HPMA copolymer-DOX-OV-TL16 [P-(GFLG)-DOX-Ab]

**Figure 4.**
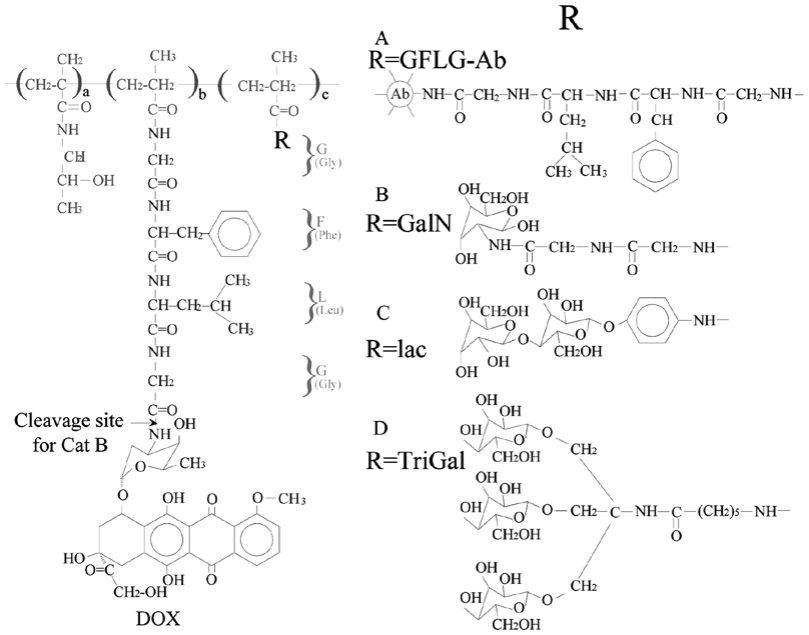
General chemical structures of (A) P-(GFLG)-DOX-Ab, (B) P-(GFLG)-DOX-GalN, (C) P-(GFLG)-DOX-lac and (D) P-(GFLG)-DOX-TriGal, in which DOX is conjugated with HPMA, by the tetrapeptide linker, Gly-Phe-Leu-Gly (GFLG).

P-(GFLG)-DOX-Ab (270,000 g/mol, 3.3 wt% DOX; Fig. 4A) is recognized by the OA3 antigen, which plays a role in membrane transport and/or signal transduction for its multimembrane-spanning domain structure (59,79–81). The P-(GFLG)-DOX-Ab is rapidly absorbed by OVCAR-3 cells and transported into their lysosomal compartment. DOX is subsequently released from the conjugate at the site with a degradable GFLG spacer, diffused via the lysosomal membrane and accumulates in the cell nuclei (80). Preliminary data on the relative retention of DOX in MDR (A2780/AD) cells have indicated a higher intracellular DOX concentration after incubation with HPMA copolymer-DOX conjugate compared with free DOX (59).

### HPMA copolymer-Gly-Phe-Lys-Gly-DOX-N-acylated galactosamine [P-(GFLG)-DOX-GalN]

P-(GFLG)-DOX-GalN (25,000 g/mol; Fig. 4B), contains N-acylated galactosamine (GalN), which was designed to be recognized by ASGPR in HepG2 human hepatocellular carcinoma cells (59,82) and individual members of the galectin family (e.g., galectin-3) in human colon adenocarcinoma (83,84). Galectin-3 is expressed in normal tissues and highly expressed in neoplastic tissues (85–87); although the exact opposite has been shown to occur (88,89). In SW-480 and SW-620 cells, the presence of galectin-3 on the cell surface has been demonstrated by flow cytometry; however, it has not been detected on the surface of Colo-205 cells. The cellular cytotoxicity of P-(GFLG)-DOXGalN determined by MTT assay has been shown to be ∼10-fold higher than P-GFLG-DOX and 10-fold higher in Colo-205 cells than in SW-480 and SW-620 cells (90). This suggests the participation of other galectins, such as galectin-1, -4, -7 or -8, in P-(GFLG)-DOX-GalN targeting.

### Lactose-containing HPMA copolymer-doxorubicin conjugate [P-(GFLG-DOX)-lac]

P-(GFLG-DOX)-lac (Fig. 4C) (90), can also be biorecognized by galectin-3 on the surface of colon cancer cells. The *in vitro* cytoxicity determined by MTT assay is higher than that of the non-glycosylated P-(GFLG)-DOX product and almost 1,000-fold lower than that of free DOX in HepG2 human hepatocellular carcinoma cells and Colo-205, SW-480 and SW-620 colon adenocarcinoma cells.

### Trivalent galactose-containing HPMA copolymerdoxorubicin conjugate [P-(GFLG-DOX)-TriGal]

P-(GFLG-DOX)-TriGal (Fig. 4D) contains trivalent galactose, which can also be biorecognized by galectin-3 on the surface of colon adenocarcinoma cells. The cytotoxicity of the P-(GFLGDOX)-TriGal has been shown to be at least 10-fold higher than that of the non-glycosylated P-(GFLG)-DOX product in Colo-205, SW-480 and SW-620 colon adenocarcinoma cells (90).

### N-Methacryloyl-glycyl)-dl-phenylalanyl-leucyl-glycyl-DOX (Ma-GFLG-DOX)

Ma-GFLG-DOX contains the tetrapeptide, Gly-Phe-Leu-Gly (91,92). It remains quite stable in buffer at pH 7.4 (model of the bloodstream), but releases DOX either under mild acidic conditions or in the presence of Cat B (rich in the tumor microenvironment).

### PAMAM dendrimers (D-NH2)-Gly-Phe-Leu-Gly-HPMA-doxorubicin (D2-GFLG-P-DOX)

D2-GFLG-P-DOX (215,000 g/mol, 9.2 wt% DOX), which is attached to DOX via a pH-sensitive hydrazone bond (91,93), was prepared by grafting the semitelechelic HPMA copolymers, which have Mws below the renal threshold, onto a PAMAM dendrimer core via a biodegradable linkage GFLG oligopeptide. An *in vitro* study using phosphate buffers at pH 5.0 or 7.4 at 37°C (hydrazone conjugates) and in a Cat B-containing (5×10^−7^ M) phosphate buffer at 37°C (amide conjugates) showed that the presence of Cat B increased the rate of DOX release (91).

### HMW1D

HMW1D (115,000 g/mol, 7.4 wt% DOX), a branched polymer prodrug, contains water-soluble polymer drug carriers, HPMA copolymers, and a biodegradable oligopeptide sequence, GFLG, linking shorter polymer chains (Mw, 20,000 g/mol) into a high-Mw structure (Mw, 110,000 g/mol) to enhance the passive accumulation of the drug by increasing its Mw. An *in vitro* study showed that this pH-sensitive prodrug (HMW1D) can be degraded by Cat B (5×10^−7^ M), 37°C, pH 6.0 (93).

### TET1D

**Figure 5.**
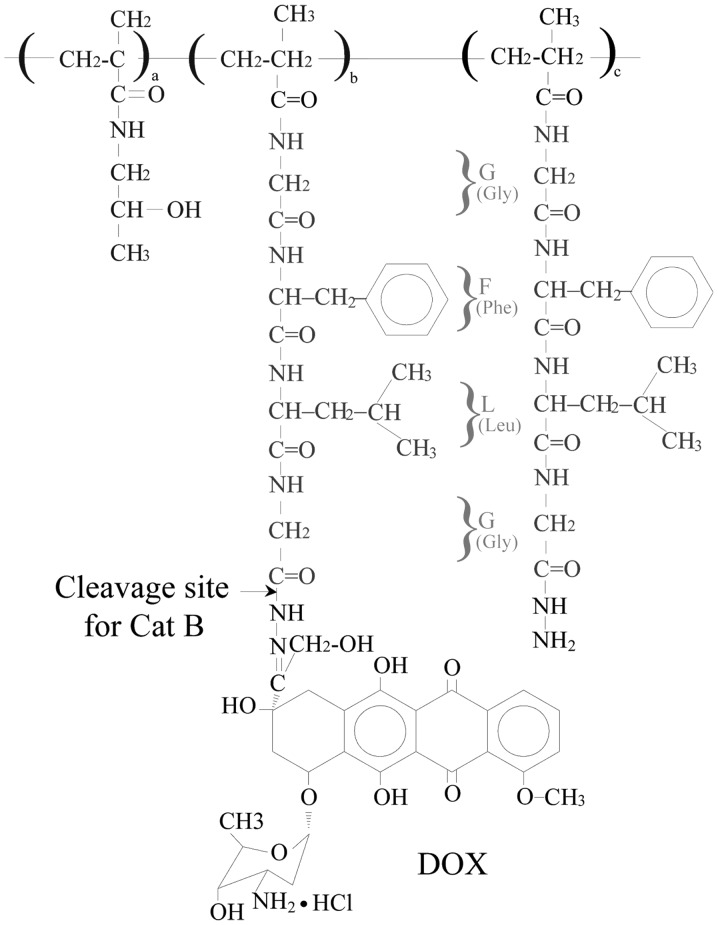
Structure of TET1D, in which DOX is combined with the tetrapep-tide, Gly-Phe-Leu-Gly (GFLG).

TET1D (19,600 g/mol, 10.5 wt% DOX; Fig. 5) (93) a non-targeted polymer-bound doxorubicin conjugate, contains a hydrazone bond, which significantly improves the rate of DOX release, compared with that of classical HPMA polymer prodrugs bearing DOX attached via amide bonds limited to maximum 8–9 wt%. An *in vitro* study using T-splenocytes and mouse EL-4 T cell lymphoma cells showed that the toxicity of TET1D is much higher compared with that of similar classic conjugates and an *in vivo* study using EL4 T cell lymphoma mice C57BL/10 showed that the antitumor activity was also significantly increased. An *in vitro* study showed that TET1D can be cleaved by Cat B; however, Cat B is not essential in the release of DOX, for it also contains a pH-sensitive spacer which is stable under physiological conditions (pH 7.4, e.g., blood) and hydrolytically degradable in a mild acidic environment (pH 5.0, e.g., endosome) (93).

### DOX prodrugs containing the tetrapeptide, Ala-Leu-Ala-Leu 6-Maleimidocaproic acid-Arg-Arg-Ala-Leu-Ala-Leu-DOX (EMC-Arg-Arg-Ala-Leu-Ala-Leu-DOX)

EMC-Arg-Arg-Ala-Leu-Ala-Leu-DOX bears maleimide (94), which can rapidly and selectively react in situ with the cysteine-34 position of circulating albumin after intravenous administration and release the drug at the tumor site (95,96). Albumin is a promising drug carrier due to its passive accumulation in solid tumors, which have a high metabolic turnover, angiogenesis, hypervasculature, defective vascular architecture and impaired lymphatic drainage (97). Albumin has non-toxic, non-immunogenic, biocompatible and biodegradable properties (98) and has demonstrated preferential tumor uptake in various tumor xenograft animal models (99). The antitumor efficacy of EMC-Arg-Arg-Ala-Leu-Ala-Leu-DOX has been shown to be comparable to that of free DOX in a M-3366 breast cancer xenograft model at equivalent doses (94). Moreover, the albumin-binding DOX prodrug, DOX-EMCH (INNO-206), has been examined in clinical trials (100,101).

### DOX prodrugs containing the dipeptide, Phe-Lys Ac-Phe-Lys-PABC-DOX

**Figure 6.**
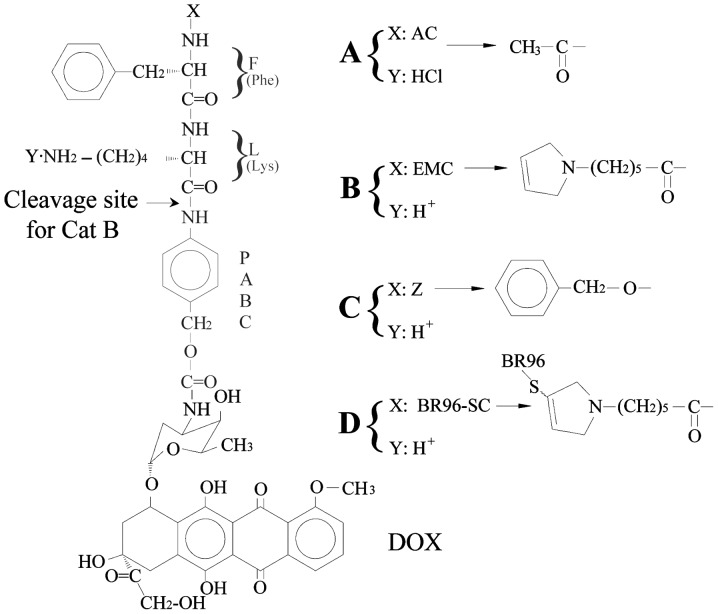
Structure of (A) Ac-Phe-Lys-PABC-DOX, (B) EMC-Phe-Lys-PABC-DOX, (C) Z-Phe-Lys-PABC-DOX and (D) BR96-SC-Phe-Lys-PABC-DOX, in which DOX is linked to the self-immolative spacer, para-aminobenzyloxycarbonyl (PABC) and the dipeptide, Phe-Lys (FL).

**Figure 7.**
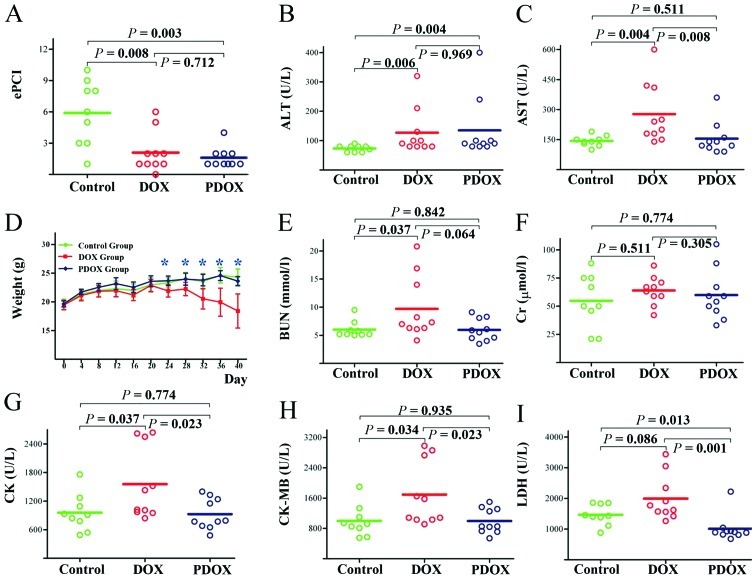
Cat B-cleavable prodrug Ac-Phe-Lys-PABC-DOX (PDOX) enhances treatment efficacy and reduces toxicity in treating gastric cancer with peritoneal carcinomatosis [modified from a previous study (12)]. (A) Effects of DOX and PDOX on a peritoneal carcinomatosis model are shown with the detailed experimental peritoneal carcinomatosis index (ePCI) score; both DOX and PDOX significantly reduced the ePCI. PDOX reduced general toxicity and toxicity to the liver, kidney and the heart in particular. (D) Nude mice in the PDOX group had similar body weights to those in the control group throughout the study period, while nude mice in the DOX group showed a progressive decrease in body weight after 4 doses of intraperitoneal injection. Effects of PDOX and DOX on major liver and renal function parameters are shown in (B) ALT, (C) AST, (E) BUN and (F) Cr. PDOX significantly decreased hepatotoxicity compared with DOX in terms of AST. PDOX significantly decreased myocardial toxicity compared with DOX by reducing (G) CK, (H) CK-MB and (I) LDH. ALT, alanine aminotransferase; AST, aspartate aminotransferase; BUN, blood urea nitrogen; Cr, creatinine; CK, creatine kinase; CK-MB, creatine kinase-MB isoenzyme; LDH, lactate dehydrogenase.

Ac-Phe-Lys-PABC-DOX (PDOX, 1045.5 g/mol, 52.0% DOX, Fig. 6A) contains the dipeptide, Phe-Lys, which is specific for Cat B and the self-immolative spacer, PABC (12,102–104). An *in vivo* study using a nude mice model of gastric cancer with peritoneal carcinomatosis showed that, compared with free DOX, PDOX (16 mg/kg, twice that of DOX in terms of equal molecular content) produced better antitumor effects in terms of experimental peritoneal carcinomatosis index (ePCI) (Fig. 7A) and body weight (Fig. 7D), and reduced liver (Fig. 7B and C), kidney (Fig. 7E and F) and heart (Fig. 7G–I) toxicities (12).

### ε-maleimidocaproic acid-Phe-Lys-PABC-DOX (EMC-Phe-Lys-PABC-DOX)

EMC-Phe-Lys-PABC-DOX (Fig. 6B) (2,18,104) has exhibited dramatic differences in antitumor activity between *in vitro* and *in vivo* studies. An *in vitro* cytotoxicity study using the pancreatic tumor cell line, AsPC1 LN, and the melanoma cancer cell line, MDA-MB-231 LN, showed that DOX was ∼6-fold more active than the prodrug. However, an *in vivo* study using a breast cancer xeno-graft nude mice model of MDA-MB-435 cells showed that the prodrug exhibited superior antitumor activity (tumor size, 15% of that in nude mice treated with the vehicle) compared to DOX (tumor size, 49% of that in nude mice treated with the vehicle) in an equitoxic comparison (2).

### PG-Phe-Lys-DOX

Hyperbranched polyglycerol-Phe-Lys-DOX (PG-Phe-Lys-DOX, 45% DOX) (18,41,105), contains the dipeptide, Phe-Lys, and hyperbranched polyglycerol. The drug release of the conjugates suggested an effective cleavage of PG-Phe-Lys-DOX and release of DOX in the presence of Cat B. The IC_50_ of PG-Phe-Lys-DOX in the breast cancer cell line, MDA-MB-231, and the pancreatic carcinoma cell line, AsPC1, was 1.10±0.4 and 2.4±0.6 *μ*M, respectively, both of which were lower than that of free DOX (105).

### Z-Phe-Lys-PABC-DOX

Benzyloxycarbonyl-Phe-Lys-PABC-DOX (Z-Phe-Lys-PABC-DOX; Fig. 6C), is stable in human plasma and rapidly releases DOX in the presence of Cat B at 37°C, pH 5.0 (half-life, 8 min), which is 30-fold faster than that of the Val-Cit conjugate. On the other hand, the release rate is significantly faster than Z-Phe-Lys-DOX, suggesting that a self immolative spacer, such as PABC, is helpful for DOX release from conjugates (104).

### BR96-SC-Phe-Lys-PABC-DOX

BR96-SC-Phe-Lys-PABC-DOX (Fig. 6D) contains the chimeric monoclonal antibody, BR96, that binds specifically to a Lewis^y^-related, tumor-associated antigen expressed on the surface of many human carcinoma cells. An *in vitro* study using human carcinomal cell lines expressing varying levels of the BR96 antigen showed that the cytotoxicity of BR96-Phe-Lys-PABC-DOX was directly related to the level of antigen expression on the cell membrane: the higher level of BR96 antigen, the higher the sensitivity to BR96-Phe-Lys-PABC-DOX. The cytotoxicity of BR96-Phe-Lys-PABC-DOX in high BR96 antigen-expressing cell lines is higher than that of the non-binding IgG-SC-Phe-Lys-PABC-DOX conjugate (>220-fold), confirming its BR96 antigen specificity (104).

### Other DOX prodrugs containing dipeptides

Dubowchik *et al*(104) and de Groot *et al*(106) synthesized a series of other DOX prodrugs containing the dipeptides, Phe-Lys, Ala-Lys or Phe-Arg, including Z-Phe-Lys-PABC-DOX•HCl, MC-Phe-Lys(MMT)-PABC-DOX, MC-Phe-Lys-PABC-DOX•Cl_2_CHCO_2_H, Z-Phe-Lys(alloc)-DOX, Z-Phe-Lys-DOX•HCl, Z-Ala-Lys(alloc)-PABC-DOX, Z-Ala-Lys-PABC-DOX•HCl, Z-Phe-Arg(NO_2_)-PABC-DOX, Z-Phe-Arg(Ts)-PABC-DOX, Fmoc-Phe-Lys(Aloc)-PABC-DOX and H-Phe-Lys(Aloc)-PABC-DOX. However, data regarding their antitumor activity are lacking.

## 5. Conclusions

Over the past few decades, significant efforts have been made to develop antitumor prodrugs with increased efficacy and decreased toxicity. Numerous DOX prodrugs have been synthesized by structure modification strategies. Cat B-cleavable DOX prodrugs release the free drugs in the presence of Cat B and in a subacidic environment. A number of *in vitro* cancer cell studies and *in vivo* tumor xenograft studies have demonstrated Cat B-cleavable DOX prodrugs to be less toxic *in vitro* and more effective *in vivo*, demonstrating the role of Cat B.

However, there remain many challenges and questions. The majority of the studies mentioned in this review are in a very early preclinical stage with little information on physicochemical properties, cytotoxicity and antitumor efficacy in tumor cells and xenografts. The subcellular distribution of the prodrugs, the free drugs released and the antitumor mechanisms remain unclear. Further studies are warranted and should focus on preclinical and clinical evaluation of existing prodrugs, rather than synthesizing novel drug candidates in this field.

## Figures and Tables

**Table I.** List of Cat B-cleavable DOX prodrugs.

Name	Biodegradable spacer	Mw (g/mol)	DOX proportion	Current status	MTD	Refs.
DOX	None	543.5	100%	Clinical therapy	60–80 mg/m^2^	(69)
PK1	Gly-Phe-Leu-Gly	30,000	8 (wt%)	Phase II	320 mg/m^2^	(36,38,39, 61–71)
PK2	Gly-Phe-Leu-Gly	27,000	8 (wt%)	Phase I/II	160 mg/m^2^	(10,11,40, 41,72–76)
P-DOX	Gly-Phe-Leu-Gly	22,000–1,230,000	NA	Preclinical	ND	26,77,78
P-(GFLG)-DOX-Ab	Gly-Phe-Leu-Gly	270,000	3.3 (wt%)	Preclinical	ND	(59,79–81)
P-(GFLG-DOX)-GalN	Gly-Phe-Leu-Gly	25,000/46,000	5.6/1.5 (wt%)	Preclinical	ND	(59,82,90)
P-(GFLG-DOX)-Lac	Gly-Phe-Leu-Gly	20,000–32,000	1.4 mol%	Preclinical	ND	(90)
P-(GFLG-DOX)-TriGal	Gly-Phe-Leu-Gly	20,000–32,000	2.1 mol%	Preclinical	ND	(90)
Ma-GFLG-DOX	Gly-Phe-Leu-Gly	NA	NA	Preclinical	ND	(91,92)
D2-GFLG-P(DOX^H^)	Gly-Phe-Leu-Gly	215,000	9.2 (wt%)	Preclinical	ND	(91,93)
HMW1D	Gly-Phe-Leu-Gly	115,000	7.4 (wt%)	Preclinical	ND	(93)
TET1D	Gly-Phe-Leu-Gly	19,600	10.5 (wt%)	Preclinical	ND	(93)
EMC-Arg-Arg-Ala-Leu-Ala-Leu-DOX	Ala-Leu-Ala-Leu	NA	NA	Preclinical	ND	(94–96)
Ac-Phe-Lys-PABC-DOX	Phe-Lys	1045.5	52.0 (wt%)	Preclinical	ND	(12)
EMC-Phe-Lys-PABC-DOX	Phe-Lys	1133	50.0 (wt%)	Preclinical	ND	(2,18,104)
PG-Phe-Lys-DOX	Phe-Lys	1207.8	45.0 (wt%)	Preclinical	ND	(18,41,105)
Z-Phe-Lys-PABC-DOX	Phe-Lys	1074.0	50.6 (wt%)	Preclinical	ND	(104)
BR96-SC-Phe-Lys-PABC-DOX	Phe-Lys	NA	NA	Preclinical	ND	(104)

Mw, molecular weight; DOX, doxorubicin; MTD, maximum tolerated dose; NA, not available; ND, not done.

## Abbreviations:

Cat B: cathepsin B
DOX: doxorubicin
HPMA: N-(2-hydroxypropyl)methacrylamide
PK1: HPMA copolymer-Gly-Phe-Leu-Gly-doxorubicin
PK2: galactosamine-targeted poly(HPMA)-doxorubicin
P-DOX: HPMA copolymer-doxorubicin conjugates
P-(GFLG)-DOX-Ab: HPMA copolymer-DOX-OV-TL16
P-(GFLG)-DOX-GalN: HPMA copolymer-Gly-Phe-Lys-Gly-DOX-N-acylated galactosamine
P-(GFLG-DOX)-lac: lactose-containing HPMA copolymer-doxorubicin conjugate
P-(GFLG-DOX)-TriGal: trivalent galactose-containing HPMA copolymer-doxorubicin conjugate
Ma-GFLG-DOX: (N-methacryloyl-glycyl)-dl-phenylalanyl-leucylglycyl-DOX
D2-GFLG-P-DOX: PAMAM dendrimers (D-NH2)-Gly-Phe-Leu-Gly-HPMA-doxorubicin
EMC-Arg-Arg-Ala-Leu-Ala-Leu-DOX: 6-maleimidocaproic acid-Arg-Arg-Ala-Leu-Ala-Leu-DOX
EMC-Phe-Lys-PABC-DOX: ε-maleimidocaproic acid-Phe-Lys-PABC-DOX
PG-Phe-Lys-DOX: hyperbranched polyglycerol-Phe-Lys-DOX
Z-Phe-Lys-PABC-DOX: benzyloxycarbonyl-Phe-Lys-PABC-DOX
